# Optimization of FK-506 production in *Streptomyces tsukubaensis* by modulation of Crp-mediated regulation

**DOI:** 10.1007/s00253-023-12473-9

**Published:** 2023-03-23

**Authors:** Susann Schulz, Håvard Sletta, Kristin Fløgstad Degnes, Sergii Krysenko, Alicia Williams, Silje Malene Olsen, Kai Vernstad, Agnieszka Mitulski, Wolfgang Wohlleben

**Affiliations:** 1 Department of Microbiology and Biotechnology, Interfaculty Institute of Microbiology and Infection Medicine Tübingen (IMIT), University of Tübingen, Auf der Morgenstelle 28, 72076 Tübingen, Germany; 2 Present Address: Novartis AG, Stein, Switzerland; 3 Department of Biotechnology and Nanomedicine, SINTEF Industry, Richard Birkelands vei 3, Trondheim, Norway; 4 Cluster of Excellence ‘Controlling Microbes to Fight Infections’, University of Tübingen, Auf der Morgenstelle 28, 72076 Tübingen, Germany; 5 Present Address: Valent BioSciences, 1910 Innovation Wy Suite 100, Libertyville, IL 60048 USA; 6 Department of Biotechnology and Nanomedicine, SINTEF Industry, Sem Sælands veg 2a, Trondheim, Norway

**Keywords:** Pipecolic acid, FK-506 regulation, Tacrolimus, Crp regulator, *S. tsukubaensis*

## Abstract

**Abstract:**

FK-506 is a potent immunosuppressive macrocyclic polyketide with growing pharmaceutical interest, produced by *Streptomyces tsukubaensis*. However, due to low levels synthesized by the wild-type strain, biotechnological production of FK-506 is rather limited. Optimization strategies to enhance the productivity of *S. tsukubaensis* by means of genetic engineering have been established. In this work primarily global regulatory aspects with respect to the FK-506 biosynthesis have been investigated with the focus on the global Crp (cAMP receptor protein) regulator. In expression analyses and protein-DNA interaction studies, the role of Crp during FK-506 biosynthesis was elucidated. Overexpression of Crp resulted in two-fold enhancement of FK-506 production in *S. tsukubaensis* under laboratory conditions. Further optimizations using fermentors proved that the strategy described in this study can be transferred to industrial scale, presenting a new approach for biotechnological FK-506 production.

**Key Points:**

• *The role of the global Crp (cAMP receptor protein) regulator for FK-506 biosynthesis in S. tsukubaensis was demonstrated*

• *Crp overexpression in S. tsukubaensis was applied as an optimization strategy to enhance FK-506 and FK-520 production resulting in two-fold yield increase*

**Supplementary Information:**

The online version contains supplementary material available at 10.1007/s00253-023-12473-9.

## Introduction

Tacrolimus, also known as FK-506, is an immunosuppressant isolated from *Streptomyces tsukubaensis* (Kino et al. 1987). Ascomycin (also, FK-520) is an ethyl analog of FK-506 isolated from *Streptomyces hygroscopicus* subsp. *ascomyceticus* with strong immunosuppressant properties (Andexer et al. 2011). The FK-506 structure belongs to an amide bond–containing macrolide family with members that include its naturally occurring analogs FK-520 and rapamycin. The structure determination showed that FK-506 is a strongly hydrophobic cyclic macrolactone with a molecular weight of 822.05 daltons (Tanaka et al. 1987). The chemical structure of FK-506 (C_44_H_69_NO_12_H_2_O) consists of a hemiketal-masked α,ß-diketoamide, which is incorporated into a 23-membered ring. The non-proteinogenic amino acid pipecolic acid is the only peptide portion of the molecule. Over the years, different *Streptomyces* strains have been identified as FK-506 and FK-520 producers in addition to *S. tsukubaensis* (Barreiro and Martínez-Castro 2014).

FK-506/FK-520 play an important role in organ transplantation (Trede et al. 1997; McCormack and Keating 2006), dermatology (Remitz and Reitamo 2009), the treatment of autoimmune (Akimoto et al. 2008), and viral (Karpas et al. 1992; Reis et al. 2006) diseases as well as of hypersensitivity reactions. FK-506 is not only a potent immunosuppressant; new studies have demonstrated that this compound has also a neuroprotective (Furuichi et al. 2003; Yousuf et al. 2011) and antifungal activity (Sugita et al. 2005; Jung and Yoon 2020). Even in the treatment of infections caused by the coronavirus, a combination of anti-inflammatory corticosteroids and FK-506 was shown to be successful (Russell et al. 2020).

The immunosuppressive mechanisms of FK-506 and FK-520 are distinct. They exert their effect through the suppression of the adaptive immune response by inhibition of T and B cell proliferation as well as inhibition of the production of interleukin-2 (IL-2), cytokines such as interleukin-3 (IL-3), interferon-γ (INF-γ), and the tumor necrosis factor-α (TNF-α) (Fantini et al. 2006). In recent years, FK-506 has become increasingly important as an alternative to the established drug cyclosporine (Liu et al. 1991; Jiang and Kobayashi 1999) in clinical therapy for the prevention of rejection reactions in transplantation of kidney (Laskow et al. 1998; Berloco et al. 2001), liver (McDiarmid 1998), lungs (Treede et al. 2001), and heart (Crespo-Leiro et al. 2002) and in the treatment of autoimmune diseases (Singer and McCune 1998). While FK-506 has similar immunosuppressive properties to the established drug cyclosporin, FK-520 produces a more selective immunomodulatory effect. One problem in FK-506 fermentation is the fact that undesired ascomycin (FK-520) is also produced as an impurity lowering the final yield of FK-506 and causing significant additional costs to the downstream isolation processes of FK-506 (LEK Pharmaceuticals, Process for Preparation of Tacrolimus, European Patent Office, EP 2272963 A1, 12.01, 2011).

The FK-506 biosynthetic pathway, identified by genome sequencing of the FK-506 producer *S. tsukubaensis*, was completely elucidated (Motamedi et al. 1996; Barreiro et al. 2012). Complete biosynthesis of FK-520 has been described in *S. hygroscopicus* var. *ascomyceticus* (ATCC 14891) (Wu et al. 2000). The core of the FK-506 biosynthesis machinery, identical to that of FK-520, consists of a hybrid PKS-NRPS system, involving genes *fkbB*, *fkbC*, *fkbA*, and *fkbP*. The polyketide synthases (PKS) FkbA, FkbB, and FkbC catalyze the condensation of an unusual starter unit derived from the shikimic acid pathway (4,5-dihydroxycyclo-1-enecarboxylic acid (DHCHC)) with ten extender units (two malonyl-CoA, five methylmalonyl-CoA, two methoxymalonyl-CoA, and one allylmalonyl-CoA). Afterward, the non-ribosomal peptide synthetase (NRPS) FkbP incorporates pipecolic acid derived from L-lysine into the polyketide chain that closes the ring structure in a final cyclization step (Andexer et al. 2011; Motamedi et al. 1997; Motamedi and Shafiee 1998; Mo et al. 2011; Goranovič et al. 2010). The immature macrolactone is further processed by post-PKS tailoring enzymes resulting in the final compound FK-506 (Motamedi et al. 1996; Chen et al. 2012). Interestingly, two types of tacrolimus biosynthesis gene clusters have been found in producer strains (Goranovič et al. 2012). The first type consists of a reduced set of genes (e.g., represented in *Streptomyces tacrolimicus* and *Streptomyces kanamyceticus* KCTC 9225). The second type consists of a complete set of genes, which contains 5 additional genes in the 5′-region of the *fkbG* gene (*allMNPOS*/*tcs12345*), as well as one or two additional genes in the 3′-region (*tcs6*-*fkbR*/*tcs67*) that seems to be a species-dependent feature. The biosynthesis of secondary metabolites in *Streptomyces* is characterized by high complexity. It is controlled by a hierarchical regulatory system represented by several levels. There is a complex of regulatory cascades where the highest levels of regulation (pleiotropic/global regulators) are located outside the clusters of biosynthetic genes and are involved in the regulation of the production of many secondary metabolites and morphological differentiation, while the lowest (specific regulators) are involved in the regulation of the production of a particular secondary metabolite (Martín and Liras 2010).

Secondary metabolism in streptomycetes can involve effector molecules, such as ppGpp (guanosine 3′,5′-bis(pyrophosphate) (Yang et al. 1974), cAMP (cyclic adenosine monophosphate), the γ-butyrolactones (Gräfe 1989; Horinouchi and Beppu 1992), and transcriptional regulators like the cAMP receptor protein (Crp), also known under the name CAP (catabolite activator protein). Crp belongs to the superfamily of Crp/Fnr- (fumarate nitrate reductase) transcriptional regulators (van Wezel and McDowall 2011). Although the exact molecular mechanism of the mode of action of Crp on secondary metabolite production is not understood, it constitutes an option to interfere in metabolic processes involved in natural product biosynthesis.

A lot of efforts have been reported for optimization of the biotechnological process of FK-506/FK-520 fermentation in *S. tsukubaensis* by employing different strategies including genetic approaches, strain improvement, media supplementation, metabolic and protein engineering, as well as combinational strategies (Xia et al. 2013; Ban et al. 2016; Wu et al. 2021b). Recently, a new optimization strategy has been presented that combines the enhancement of the precursor supply for L-lysine with the improvement of pipecolic acid formation in *S. tsukubaensis* (Schulz et al. 2022). But the aspect of increasing the FK-506 yield by manipulation of regulatory steps at a global level remained uninvestigated, although the effects of phosphate and N-acetylglucosamine for FK-506 biosynthesis have been studied previously (Martín 2004; Martínez-Castro et al. 2013; Ordóñez-Robles et al. 2018). Three different regulatory genes were previously characterized in the FK-506 biosynthesis gene cluster: *allN*, *fkbN*, and *fkbR* (Mo et al. 2012; Barreiro and Martínez-Castro 2014; Ordóñez-Robles et al. 2018). Similarly, in the FK-520 biosynthetic gene cluster of *S. hygroscopicus* ATCC 14891, the regulatory genes *fkbN* and *fkbR* were identified (Yu et al. 2019). The regulator FkbN belongs to the group of LAL (large ATP-binding regulators of the LuxR family) transcriptional regulators. FkbN has been shown to be active as a positive regulator of the FK506 biosynthesis (Santos et al. 2012; Mo et al. 2012). The *fkbR* gene codes for a regulator from the family of the LysR-type transcriptional regulators. FkbR has been demonstrated to be a positive regulator of FK-506 biosynthetic gene cluster by Maddocks and Oyston (2008) and Goranovič et al. (2012). In contrast, it has been reported that FkbR has a negative regulatory effect in the FK-506 biosynthesis in the alternative FK-506 producer *Streptomyces *sp. KCTC 11604BP (Mo et al. 2012). This discrepancy is a mystery because of the FK-506 biosynthesis gene clusters from *Streptomyces *sp. KCTC 11604BP and *S. tsukubaensis* NRRL 18488 demonstrate high similarity (Mo et al. 2012). Nevertheless, these results suggest that possibly different regulatory mechanisms of the biosynthetic pathway can exist in different *Streptomyces* strains (Barreiro and Martínez-Castro 2014). The *allN* gene codes for a putative regulator from the AsnC family of transcriptional regulators (Barreiro et al. 2012). However, no function could be determined so far (Goranovič et al. 2012).

Activation of Crp by cAMP binding results in the dimerization of this complex and in a conformational change that leads to the activation of the Crp C-terminal DNA binding domain (Berg and von Hippel 1988; Kim et al. 1992). The Crp-cAMP complex can act as an activator or as a repressor (Mori and Aiba 1985; Kolb et al. 1993; Görke and Stülke 2008). The Crp-cAMP regulatory network is complex and affects a large number of genes from different cellular processes in different bacterial families (Körner et al. 2003; Gama-Castro et al. 2008; Gao et al. 2012).

Crp has extensively been investigated for its role in carbon catabolite repression mostly in *Escherichia coli* and *Bacillus subtilis* (Görke and Stülke 2008). In recent years, it has been shown that the regulatory network of Crp is significantly more complex and larger than expected and that Crp is involved in many cellular processes (Mao et al. 2007). In *Streptomyces coelicolor,* the deletion of the *crp* gene resulted in a significant decrease in production of the antibiotic actinorhodin, while the overexpression of this gene led to an increase in actinorhodin synthesis. In addition, the stimulation of silent antibiotic clusters by heterologous expression of the *crp* gene from *S. coelicolor* in various actinobacteria was demonstrated (Gao et al. 2012). Furthermore, a role of Crp in the morphological differentiation and sporulation in *S. coelicolor* has been proved (Süsstrunk et al. 1998; Derouaux et al. 2004b; Piette et al. 2005).

In *Streptomyces*, transcriptional regulators from the Crp/Fnr family are widespread (Gao et al. 2012). In contrast to *E. coli*, no Crp-cAMP-dependent catabolite repression is present in streptomycetes. Different studies suggested that other mechanisms can be responsible for catabolite repression in streptomycetes (Angell et al. 1992; Kwakman and Postma 1994).

For the identification of Crp-associated binding sites in the genome of *S. coelicolor*, chromatin immunoprecipitation and ChIP-chip assays were performed (Gao et al. 2012). These experiments revealed 393 Crp target genes that encode transcriptional regulators or proteins involved in cellular processes. After the respective sequences were analyzed, the following consensus sequences for putative Crp binding were postulated: a strong conserved one [GTG(N)_6_GNCAC] or a less conserved one [GTG(N)_6_GNGAN] (Gao et al. 2012). It has also been demonstrated that such binding boxes are conserved in secondary metabolite clusters in *S. coelicolor*.

Based on recent investigation, the regulatory network that involves Crp represents a starting point for the production optimization of pharmaceutically interesting secondary metabolites (Wu et al. 2021a). Crp was shown to be an ubiquitous protein that modulates primary metabolism and enhances precursor flux to secondary metabolite biosynthesis. In previous studies the role of Crp has been addressed. Higher levels of the transcription regulators Crp and AfsQ1 have been found after the combination of metabolically engineered secondary pathways and exogenous feeding strategies, e.g., supplementation with various nutrients such as soybean oil, that resulted in enhanced FK-506 production (Xia et al. 2013). The presence of homologs in *S. coelicolor* and other *Streptomyces* suggested a conserved role in the genus (Wu et al. 2021a). In this work, we analyzed the function of Crp in FK506 biosynthesis and applied it for increasing FK-506 production.

## Materials and methods

### Analysis of sequence motifs

For search and analysis of binding sequences of specific regulators in the genome of *S. tsukubaensis*, the MEME/MAST Suite web server was used that provides a portal for online discovery and analysis of sequence motifs representing features such as DNA binding sites and protein interaction domains (Bailey et al. 2015). Results were expressed as E-value, which is the statistical significance of the motif and is an estimate of the expected number of motifs with the given log likelihood ratio and with the same width and site count, that can be found in a similarly sized set of random sequences.

For the prediction of the isoelectric point (pI/Mw) of a protein based on the amino acid sequence input, Expasy web-tool was used. It allows the computation of the theoretical pI (isoelectric point) for entered sequences (Gasteiger et al. 2005).

### Bacterial strains and plasmids

Experiments were carried out using the *S. tsukubaensis* NRRL 18488 strain from the NRRL Culture Collection of the Agricultural Research Service (USA). All mutants were derivatives of *S. tsukubaensis* NRRL 18488. *Escherichia coli* NovaBlue strain purchased from Novagen was used for standard cloning procedures, while *E. coli* Rosetta DE2 cells served as hosts for protein expression experiments. Plasmid transfer was carried out by intergeneric conjugation between the non-methylating *E. coli* ET12567/pUZ8002 strain (MacNeil et al. 1992; Kieser et al. 2000) and *S. tsukubaensis*. The DNA from *S. tsukubaensis* NRRL 18488 was used as a template for the amplification of *crp*. The genomic DNA for the amplification of *glxR* from *Corynebacterium glutamicum* DM1730 (Seibold et al. 2006) was provided by Jörn Kalinowski (University of Bielefeld, Germany).

All strains and plasmids used in this study are listed in the Table S2.

### Media and culture conditions

Spores and mycelia preparations were performed using the ISP4 agar (Difco, Sparks, MD, USA). Initial studies of FK-506 production by *S. tsukubaensis* NRRL 18488 were performed in shake flasks containing liquid MG-2.5 mM medium optimized by Martínez-Castro et al. (2013), containing 50 g/l starch (Difco), 8.83 g/l glutamic acid, 2.5 mM KH_2_PO_4_/K_2_HPO_4_, 0.2 g/l MgSO_4_·7H_2_O, 1 mg/l CaCl_2_, 1 mg/l NaCl, 9 mg/l FeSO_4_ ·7H_2_O, 21 g/l MOPS, and 0.45 ml/l 10 × trace elements; pH 6.5 was adjusted. Fermentation was performed using a two-stage culture system. For the seed culture, a mixture of 1:1 modified YEME (3 g/l yeast extract, 5 g/l Bacto peptone, 3 g/l malt extract, 10 g/l glucose, and 340 g/l saccharose) and Tryptic Soy Broth (Difco) (Jones et al. 2013) was inoculated with spores or mycelia from an ISP4 plate and cultivated for minimum of 3 days at 28 °C and 220 rpm. In total, 100 ml of MG-2.5 mM medium was inoculated with the seed culture and further incubated at same conditions for 6 days.

Seed culture for both the microbioreactor system and the 3L fermentors was produced in 500 ml baffled shake flasks containing 100 ml of TSB:YEME (1:1) medium and 3 g of 3-mm glass beads. The cultures were harvested at OD600 = 2–4. The microbioreactor system, BioLector I, enables 48 parallel cultivations with online monitoring of biomass, pH, and dissolved oxygen (DO). The cultivations were performed in round well plates (MTP-R-48-BOH) with 48 wells, a well volume of 3.6 ml, and a filling volume of 1.2 ml. Each well in the plate was equipped with optodes for measuring DO and pH, and biomass was measured via scattered light. Microfermentations were performed using 0.5 × MG-medium with 2.5 mM phosphate (0.5 × MG-2.5 mM) (Doull & Vining 1990, Martínez-Castro et al. 2013) with 0.04% Antifoam 204 (Cat. No. A6426, Sigma) and 3% inoculum from the seed cultures. After filling, the cultivation plates were covered by a gas permeable sealing foil (F-GPR48-10) and incubated in the BioLector I with a shaking frequency of 1000 rpm (3-mm orbital amplitude) at 28 °C for 6 days. Biomass (gain 30), DO, and pH were measured every 45 min, and the humidity in the chamber was controlled at 85% RH. The fermentors (3 L Applikon with 1 L medium or 1 L DasGip with 0.7 L medium) were inoculated with 3% seed cultures. The fermentation medium used was MG-2.5 mM or 2 × MG-2.5 mM (double amount of all components except MOPS), and the fermentations were run for 140 h at 28 °C, with dissolved DO controlled at 40% of saturation. The pH of the cultivations in 2 × MG-2.5 mM medium was controlled at pH = 7.2 with 2 M HCl.

### Construction of the *S. tsukubaensis* recombinant strains

The vector pRM4 (Menges et al. 2007) was used for the construction of the *S. tsukubaensis* (pRM4) strain as well as *crp* overexpression strains *S. tsukubaensis* UT1 and *S. tsukubaensis* UT2. For this purpose, the *crp* gene from *S. tsukubaensis* or the *glxR* gene from *C. glutamicum* was amplified and cloned in the integrative pRM4 plasmid with the strong _P_*ermE* promoter. The expression plasmid pRM4, pRM4-*crp*, or pRM4-*glxR* was introduced into the *S. tsukubaensis* wild-type strain via biparental conjugation. The integration was checked by PCR and sequencing. The verified strains were used for further experiments.

### Analysis of *S. tsukubaensis* growth and FK-506 production in initial studies

The productivity of a *S. tsukubaensis* strain was usually determined as the production of FK-506 per g of cell dry weight. During the fermentation time of 7 days, each 24 h 1 ml of bacterial culture was gathered, centrifuged, washed twice with distillated water, and dried by lyophilization. For FK-506 detection, the samples from the media were mixed with equal volume of ethylacetate (1:1), stirred for 10 min, and subsequently centrifuged. FK-506 and FK-520 were detected using an optimized HPLC method. The organic phase was analyzed using an HP1090 M HPLC system equipped with a diode array detector and a thermostatic autosampler. A Zorbax Bonus RP column, 3 × 150, 5 μm (Agilent Technologies, Santa Clara, USA) constituted the stationary phase. The mobile phase system was applied with 0.1% phosphoric acid and 0.2% triethylamine as eluent A and acetonitrile with 1% tetrahydrofurane as eluent B. The flow rate was 850 μl/min, and the column temperature was set to 60 °C. The absorbance was monitored at a wavelength of 210 nm. Data sets were processed with the help of the Chemstation LC 3D, Rev. A.08.03 software from Agilent Technologies. Standards of pure FK-506 (Antibioticos SA, Leon, Spain) and FK-520 (ascomycin) (Sigma-Aldrich Chemie GmbH, Munich, Germany) were used as controls.

### Analyses of FK-506 and FK-520 (ascomycin) production in cultivations under controlled conditions

Sampling from cultures in the BioLector I was performed manually once every day by putting the system on pause and withdrawing 30 µl broth from each well into polypropylene microtiter plates while the cultivation plate was on continuous shaking in a LAF hood. The samples were immediately frozen and then lyophilized for 2 days. Extraction of the dry material was performed with 120 µl DMSO while shaking for 1 h (600–700 rpm, 3 mm orbital amplitude). The plates were centrifuged (4500 rcf) for 10 min to obtain cell-free extracts for analyses. Samples from 1 L and 3 L fermentors were prepared for quantitative analyses as follows: 1 g samples of fermentation broth were extracted with 5 ml ethyl acetate for 10 min. The ethyl acetate in 300 µl of cell-free extracts was removed by evaporation, and the dry matter was reconstituted in 900 µl DMSO prior to analysis.

The volumetric yields of tacrolimus and ascomycin were quantified using an Agilent 1290 Infinity LC-QQQ method with an analysis time of 10 min per sample (method based on a previously published method (Lechner et al. 2013)). Injection volume was 2.5 µl. Separation of compounds was performed using an Acquity UPLC BEH C18 column (50 × 2.1 mm, 1.7 μm; Waters) with water added 0.77 g/l ammonium acetate and 1 ml/l formic acid (mobile phase A) and 95:5 acetonitrile:water added 0.77 g/l ammonium acetate and 1 ml/l formic acid (mobile phase B). The gradient used was 50% mobile phase B for 1 min, with a gradual increase to 70% B during the next 7.5 min, followed by a washing step with 100% B for 0.7 min. Column temperature was 40 °C. The MS was run in positive mode with gas temperature; 160 °C, gas flow; 20 l/min, nebulizer; 3520 psi, sheath gas temperature; 250 °C, sheath gas flow; 11 l/min, capillary voltage; 3000 V, nozzle voltage; 0 V. The iFunnel parameters were high pressure RF: 1650 V, and low pressure RF: 760 V. The quantifiers were tacrolimus 821.4–768.4 Da and ascomycin 809.4–756.3 Da. The standards used to quantify the products were FK-506 (Cat. No. F4679, Sigma) and FK-520 (Cat. No. A3835, Sigma).

### Reverse transcription (RT)-PCR analysis

For the analysis of gene transcription, the *S. tsukubaensis* wild type and mutants were incubated in YEME:TSB medium. After 3 days, cells were transferred into the production medium MG and incubated for further 2, 3, or 5 days. Cells were gathered by homogenization using Precellys™ 24 (Peqlab, Erlangen, Germany). RNA isolation was carried out with the RNeasy kit (Qiagen, Hilden, Germany). RNA preparations were performed twice with DNase (Fermentas, Germany): an on-column digestion was carried out for 30 min at 24 °C, and afterwards RNA samples were treated with DNase for 1.5 h at 37 °C. RNA concentrations and quality were proved using a NanoDrop ND-1000 spectrophotometer (Thermo Fisher Scientific, Karlsruhe, Germany). cDNA from 3 µg RNA was generated with random nonamer primers (Sigma-Aldrich), reverse transcriptase, and cofactors (Fermentas). PCR reactions were performed with primers listed in Table S3. The PCR conditions were as follows: 95 °C for 5 min; 35 cycles of 95 °C for 15 s, 55–60 °C for 30 s and 72 °C for 30 s, and 72 °C for 10 min. As a positive control, cDNA was amplified from the major vegetative sigma factor (*hrdB*) transcript, which is a housekeeping gene produced constitutively. To exclude DNA contamination, negative controls were carried out by using total RNA as a template for each RT-PCR reaction.

All oligonucleotides used in this study are listed in the Table S3.

### Heterologous expression and purification of Crp and GlxR proteins

For the EMSA studies, the Crp protein from *S. tsukubaensis* was produced in the *E. coli* host. For this, the construct pYT9-*glnR*-StrepII (Tiffert et al. 2008) with the rhamnose-inducible promoter P*rham* was used. The *crp* gene was amplified by PCR with the primers CRP_StrepIINdeI/CRPpYTrewHind using the genomic DNA of *S. tsukubaensis* as a template. At the N-terminal end of the gene, a StrepII fusion tag was added using the respective primer. The PCR product was cloned into the intermediate vector pDrive and checked by sequencing. Inserts were cut out using the restriction enzymes *Nde*I and *Hin*dIII and cloned into the expression construct pYT9-*glnR-*StrepII to replace *glnR* by *crp*.

Competent *E. coli* Rosetta DE3 pLys cells with the with pYT9-*crp-*StrepII construct were used for the production of the fusion Crp protein. The purification of the StrepII-Crp protein was performed via the affinity chromatography. Eluates were checked by SDS PAGE and Western blotting. The purified ~ 25 kDa fusion protein was then concentrated to the final protein concentration and used for EMSA studies.

## Results

### *In silico* analysis of the *crp* gene and its putative binding sites

**Fig. 1:**
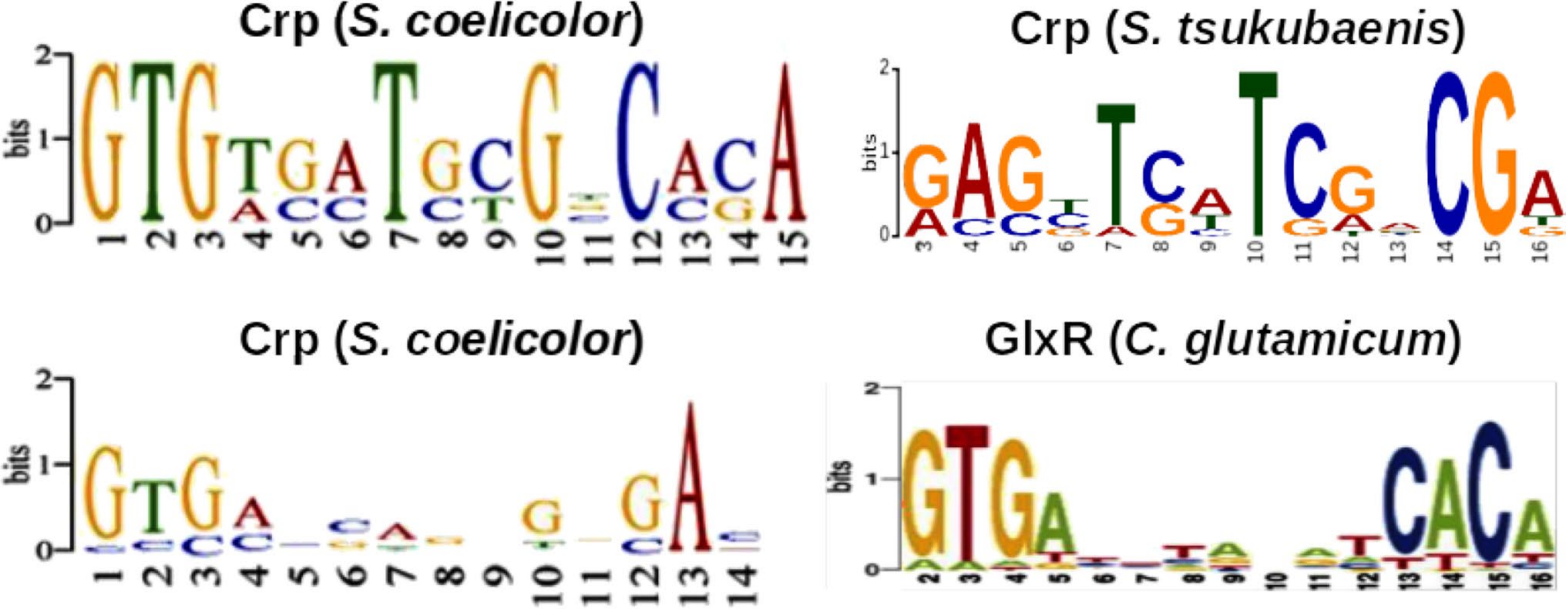
Central motifs of Crp binding sequences in *S. coelicolor, S. tsukubaensis*, and *C. glutamicum.* Comparison of the strong conserved [GTG(N)_6_GNCAC] (top panel, left) and less conserved [GTG(N)_6_GNGAN] (bottom panel, left) binding sequences of Crp in *S. coelicolor* (left) (Gao et al. 2012) with predicted binding sequences of Crp in *S. tsukubaensis* [GAG(N)_6_GNCGA] (top panel, right) (this work) and of GlxR in *C. glutamicum* [GTG(N)_8_CACN] (bottom panel, right) (Kohl et al. 2008). For the complete output of the MEME/MAST analysis, see Fig. S1

In order to investigate whether Crp (B7R87_18110) may be involved in regulatory processes in *S. tsukubaensis*, its genome was screened for the Crp binding sequences. MEME/MAST analysis revealed the presence of the sequence motif [NCGAG(N)_6_GNCGA] at 15 sites in *S. tsukubaensis* (E-value: 1.5e-003), which is similar to binding motifs reported in other *Actinobacteria*. For example, [GTG(N)_6_GNCGA] was reported for a Crp homolog SCO3571 in *S. coelicolor* (Gao et al. 2012) and [NGTG(N)_8_CACN] was reported for the Crp homologous regulator protein GlxR (Cg0350) in *C. glutamicum* (Kohl et al. 2008) (Fig. 1). In *S. tsukubaensis*, the identified in silico motif [GAG(N)_6_GNCGA] was found across the genome in the promoter and a protein-encoding region of genes for primary and secondary metabolism.

As an additional approach, the conserved Crp binding motifs reported for *S. coelicolor* were searched in promoter regions of genes in the FK-506 biosynthesis gene cluster in *S. tsukubaensis.* In the intergenic region upstream of the *fkbB* and *fkbO* genes, a Crp binding sequence GTGAGTGTCGGCGAC, which features the conserved motif [GTG(N)_6_GNCAC] from *S. coelicolor* (Gao et al. 2012), was found. A binding sequence featuring the motif [GTG(N)_6_GNGAN] is present in the upstream region of *fkbC*. Sequences similar to these conserved motifs were also detected in the coding region of the structural genes: *fkbA*, *fkbO* (at the beginning of each gene sequence); *fkbP*, *fkbB*, *fkbC* (at other positions of each gene sequence). Interestingly, a binding sequence similar to the motif [GTG(N)_6_GNCGA] is also present in the upstream region of the *fkbL* gene (GTGTTCTTCGCCGC) that encodes the lysine cyclodeaminase enzyme required for the synthesis of pipecolic acid.

In addition to the FK-506 biosynthetic gene cluster, Crp binding sequences were found in the promoter region of genes for the primary metabolism: *glnA* (GTGGCTGACCGCA), *gltB* (GTGGAACCCGGCG), and *glnII* (GTGGGAAATCGCC) (Table S1). Moreover, in the promoter region of the *crp* gene, a Crp binding sequence GTGCGTGCTGCGA, which features the conserved motif [GTG(N)_6_GNGAN], was found. In addition the *cya* gene encoding the adenylate cyclase responsible for the generation of the cyclic adenosine monophosphate (cAMP) harbors a Crp binding motif [GTG(N)_6_GNCAC] (Table S1).

### Expression analysis of FK-506 biosynthetic genes after *crp* overexpression

In order to analyze the effect of *crp* overexpression on the *crp* gene itself as well as on the transcription of the FK-506 biosynthetic genes, comparative expression analysis of genes in *S. tsukubaensis* was performed using reverse transcriptase-PCR (RT-PCR). First, the expression of the *crp* gene was analyzed in *S. tsukubaensis* wild type and *S. tsukubaensis* UT1, a derivative of *S. tsukubaensis* NRRL18488, in which *crp* was constitutively expressed under the control of the *ermE* promoter (see the “Materials and methods” section).

**Fig. 2:**
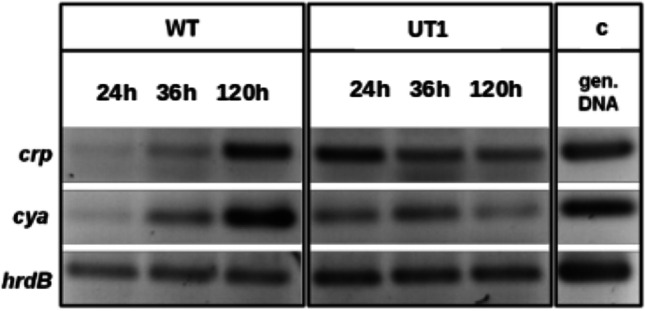
Transcriptional analysis of the *crp* and *cya* genes. RT-PCR analysis: *crp* and *cya* constitutively expressed in the *S. tsukubaensis crp* overexpression strain (UT1) in contrast to the *S. tsukubaensis* wild type (WT). *hrdB* was used as a control (c)

The enhanced expression of *crp* resulted in a high expression level of the houskeeping gene *cya*. This was demonstrated by RT-PCR: overexpression of *crp* led to the expression of *cya* as well as *crp* at all tested time points, in contrast to the wild type, which showed a strong expression of *crp* and *cya* only after 5 days of incubation (Fig. 2).

**Fig. 3:**
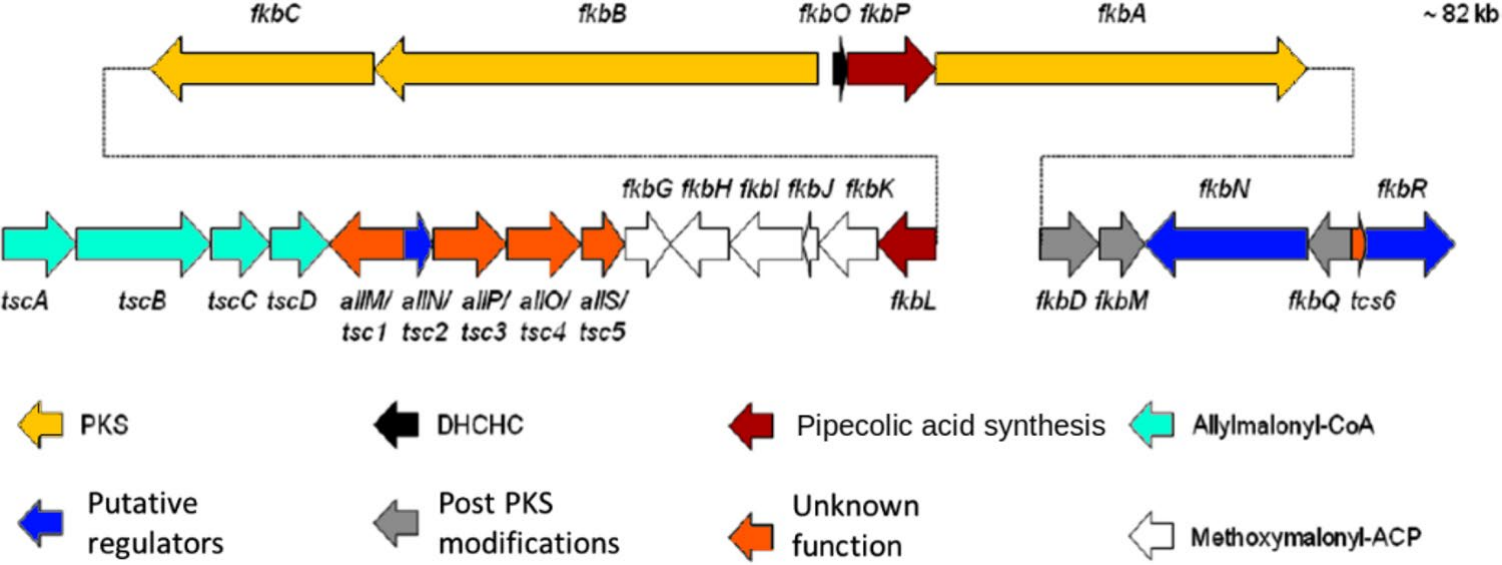
Organization of the FK-506 biosynthetic cluster (*fkb*). Genes are shown by their localization and respective functions. Modified after Ordóñez-Robles et al. (2018). PKS, polyketide synthase; DHCHC, 4,5-dihydroxycyclohex-1-enecarboxylic acid; CoA, coenzyme A

Secondly, after the verification of the influence of the *crp* overexpression on the housekeeping gene *cya*, such effects were further investigated in regard to the FK-506 biosynthetic genes. Transcriptional analysis by RT-PCR was performed for key FK-506 biosynthetic genes: *fkbC* and *fkbB* encoding polyketide synthases involved in the formation of the basic FK-506 structure, *fkbP* encoding a non-ribosomal peptide synthetase for the elongation unit, *fkbL* encoding the lysine cyclodeaminase converting lysine into pipecolate, and *fkbO* encoding a chorismatase involved in the supply of the starter unit (Fig. 3).

**Fig. 4:**
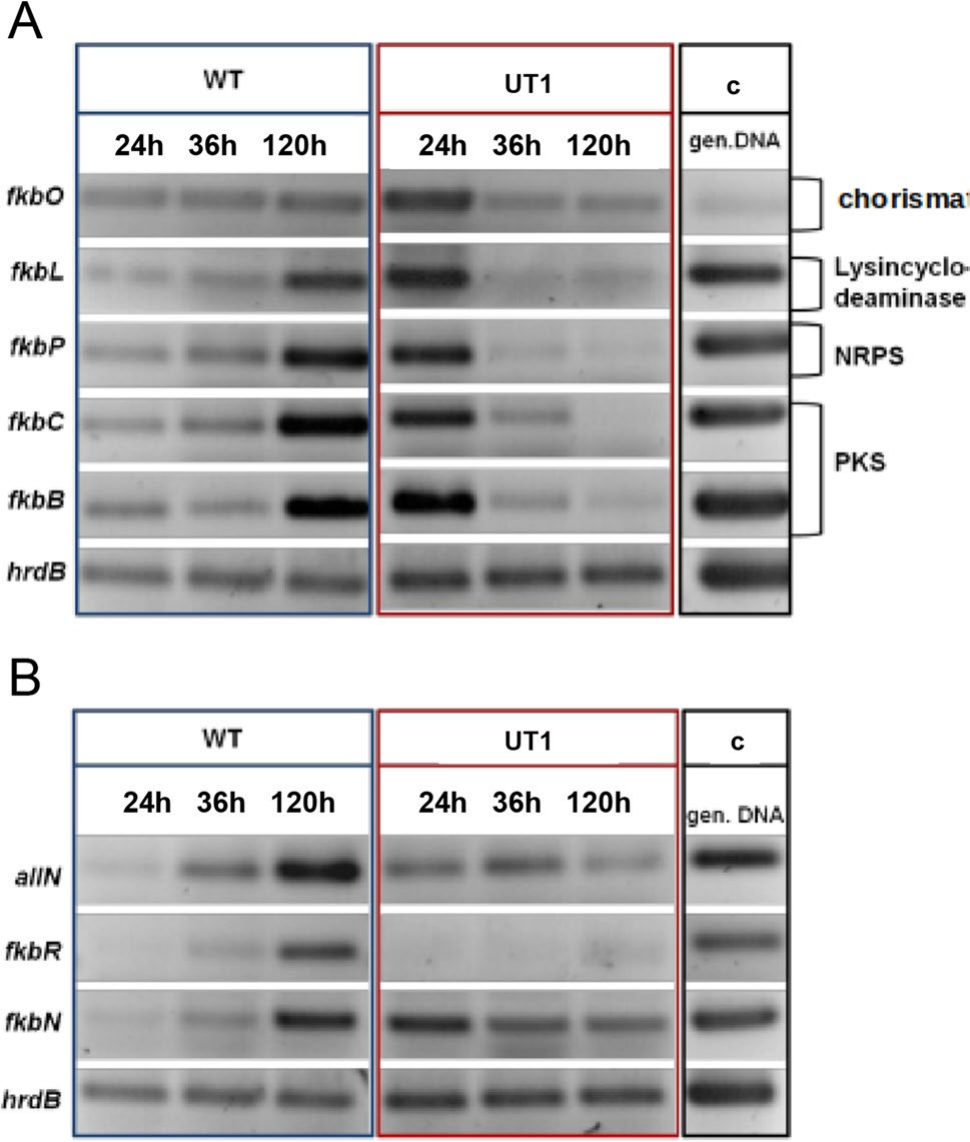
Transcriptional analysis of the FK-506 biosynthetic and regulatory genes in different *S. tsukubaensis* strains. **A** Expression profiles of FK-506 biosynthetic genes in the *S. tsukubaensis* wild type (WT) and *crp* overexpression strain (UT1). NRPS, nonribosomal peptide synthetase; PKS, polyketide synthase. **B** Expression analyses of the pathway-specific regulatory genes of the FK-506 gene cluster. As controls, genomic DNA from *S. tsukubaensis* was used and the expression of *hrdB* was determined (c)

While the expression of the structural genes in the wild type increased over time, the expression of the same genes in the *S. tsukubaensis* UT1 *crp* overexpression strain was enhanced already from the beginning and decreased over time (Fig. 4). These results suggested a direct impact of Crp on the FK-506 biosynthetic gene cluster.

It was further tested whether Crp-overexpression also has effects on the regulator genes of the FK-506 biosynthetic gene cluster. Overexpression of Crp indeed also affected *allN*, *fkbR*, and *fkbN*: *allN* demonstrated a constitutive expression; *fkbR* no transcription and *fkbN* an almost constitutive expression (Fig. 4). These results let to assume that the specific regulators AllN, FkbN, and FkbR play a subordinate role in the regulatory cascade, in which Crp is a global high-level regulator.

The overall results of the transcriptional analysis indicate that the overexpression of *crp* caused effects on the expression of FK-506 biosynthetic and regulatory genes, demonstrating a direct impact of *crp* on the FK-506 production.

### Expression analysis of genes of the nitrogen metabolism

Since it is known that also metabolic steps of the primary metabolism can limit antibiotic yields (Craney et al. 2012) and that in *S. coelicolor* the Crp regulator can interact with genes from the primary metabolism, we aimed to test the effect of Crp on the expression of such genes. Previous investigations on the role of the nitrogen metabolism in antibiotic production (Waldvogel et al. 2011; Nieselt et al. 2010) indicated that the following genes are most relevant: *amtB* encoding the ammonium transporter in an operon with *glnK* (PII protein) and *glnD* (uridylyl transferase); *gltB* encoding the glutamate synthase; *glnII* encoding the glutamine synthetase II; *glnA* encoding the glutamine synthetase I.

**Fig. 5:**
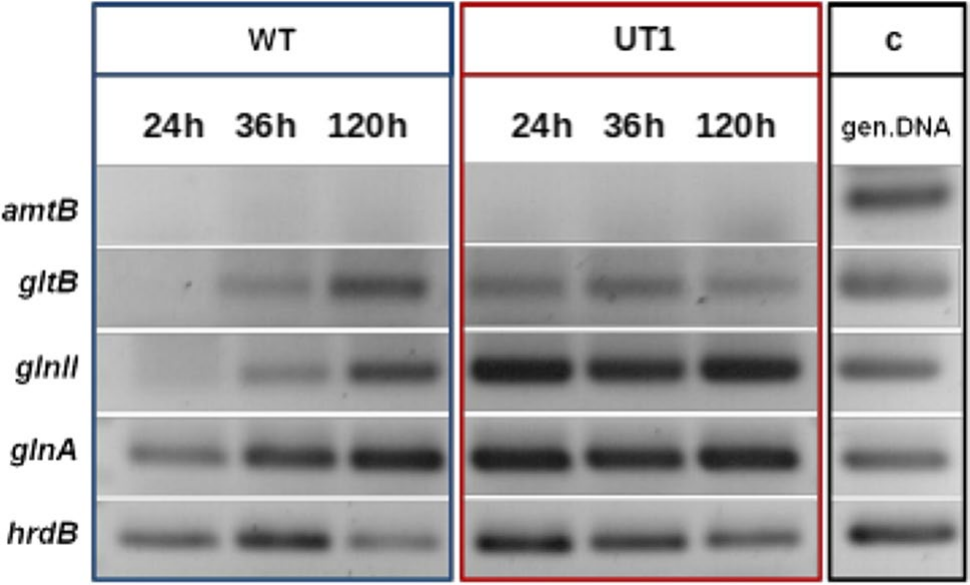
Transcriptional analysis of the genes of the primary nitrogen metabolism in different *S. tsukubaensis* strains during FK-506 production. Expression profiles of the genes of the nitrogen metabolism in the *S. tsukubaensis* wild type (WT) and in the *crp* overexpression strain (UT1). Genomic DNA from *S. tsukubaensis* as well as the *hrdB* gene were used as controls (c)

No expression of *amtB* in the *S. tsukubaensis* wild type and in the *crp* overexpression strain *S. tsukubaensis* UT1 was observed. Constitutive low expression throughout the whole cultivation period was only detected for *gltB* in *S. tsukubaensis* UT1, in contrast to a time-dependent expression in the wild type. In the wild-type strain, the expression of *gltB*, *glnA*, and *glnII* appeared after prolonged incubation. In contrast, both glutamine synthetase encoding genes *glnA* and *glnII* revealed constitutive high expression in *S. tsukubaensis* UT1 (Fig. 5).

These results are in agreement with in silico analyses. While unique Crp binding sequences in front of *gltB* (GTGGAACCCGGCG), *glnII* (GTGGGAAATCGCC), and *glnA* (GTGACGTACCCGA) were found in *S. tsukubaensis*, no Crp binding sequence was found in the promoter region of *amtB*.

The results indicate that the overexpression of *crp* causes effects not only on the expression of FK-506 biosynthetic genes, but also on the expression of specific genes of the primary nitrogen metabolism.

### Analysis of *crp* overexpression on FK-506 production in *S. tsukubaensis*

To test the influence of Crp on FK-506 yield, initial production tests with *S. tsukubaensis* wild type (WT), *S. tsukubaensis* (pRM4) with the empty integrative plasmid pRM4 and *S. tsukubaensis* UT1 were carried out in shake flasks. The *S. tsukubaensis* wild type (WT) produced ~ 1.8 mg FK-506 per g cells. The *crp* overexpression resulted in a ~ 65% increase of the FK-506 yield (3 mg/g), whereas the vector control did not reveal a significant difference to the wild type (~ 1.75 mg/g) (Fig. S2).

In parallel, time-dependent FK-506 production was monitored by HPLC analysis. The following time points were selected: 48 h (start of FK-506 production); 72 h (maximum of FK-506 production), and 120 h (end of production phase). The study of the gene expression was performed in agreement with the FK-506 production: the HPLC analysis demonstrated an increase of FK-506 production over time after *crp* overexpression (Fig. S3). Moreover, our results indicated a particularly large impact of *crp* on FK-506 yield in the early production phase.

### DNA–protein interaction studies with purified Crp protein from *S. tsukubaensis*

The identification of putative Crp binding sequences (see above) and the effects of *crp* overexpression indicate that a direct interaction of Crp with the DNA region in front of the respective genes should occur. In order to study the interaction of Crp with the identified target genes, electromobility shift assays (EMSAs) were carried out. For this purpose, a StrepII fusion tag was added at the N-terminal end of the *crp* gene. Competent *E. coli* Rosetta DE3 pLys cells with the pYT9-*crp-*StrepII construct were used for the production of the fusion Crp protein. The StrepII-Crp protein was purified using affinity chromatography. For initial tests the *cya* gene, which harbors a Crp binding site in its promoter, was selected. For the binding studies of Crp, the *cya* promoter region (a 160 bp fragment) was used. However, no band shifts could be observed (Fig. S4), which might be due to the unusual isoelectric point of the used StrepII-Crp fusion protein that is: pI = 5.79 for the native protein; pI = 5.92 for StrepII-Crp. This result is in agreement with previous unsuccessful attempts from different researchers to prove the binding of the *Streptomyces* Crp protein to potential target sequences (Derouaux et al. 2004a; Gao et al. 2012).

### DNA–protein interaction studies with the purified GlxR protein from *C. glutamicum*

In closely related *Actinobacteria* like *Corynebacterium glutamicum* ATCC 13032, no analog of a classical Crp is present. Instead, GlxR (cg0350) that resembles *S. tsukubaensis* Crp (54% identity) has been characterized as a cAMP-binding transcription regulator from the CRP/FNR protein family (Kohl et al. 2008). The DNA binding of GlxR has been proven by EMSA studies (Kohl et al. 2008) with *C. glutamicum*. Comparison of the published GlxR binding motif [GTG(N)_8_CAC] with the deduced strongly conserved Crp binding sequence [GTG(N)_6_GNCAC] in *S. coelicolor* as well as [GAG(N)_6_GNCGA] in *S. tsukubaensis* revealed high similarity (Fig. S1). We therefore speculated that GlxR can be used to test whether binding of this type of regulator to the deduced Crp binding sequences can occur, since compared to Crp, GlxR possesses a less unusual isoelectric point (pI = 6.60 for the native protein; pI = 6.95 for 6xHis-GlxR).

In order to prove the binding of the GlxR protein to the Crp target sequences in *S. tsukubaensis*, the GlxR protein was heterologously expressed as an N-terminal fusion protein (His-Tag) in *E. coli* JM109. Subsequently, His-GlxR was purified and checked by SDS-PAGE and Western blotting. The purified His-GlxR protein was then directly used for EMSA studies. Thereby, the interaction of His-GlxR with the *cya* promoter region of *S. tsukubaensis* was demonstrated indicating binding of GlxR upstream of the *cya* gene (Fig. S4).

This result showed that the GlxR regulator from *C. glutamicum* can recognize the binding sequences of Crp and can interact with *S. tsukubaensis* genes. Thus, GlxR was subsequently used to conduct interaction studies on Crp target genes in *S. tsukubaensis,* which appeared to be relevant for FK-506 production in gene expression analysis.

**Fig. 6:**
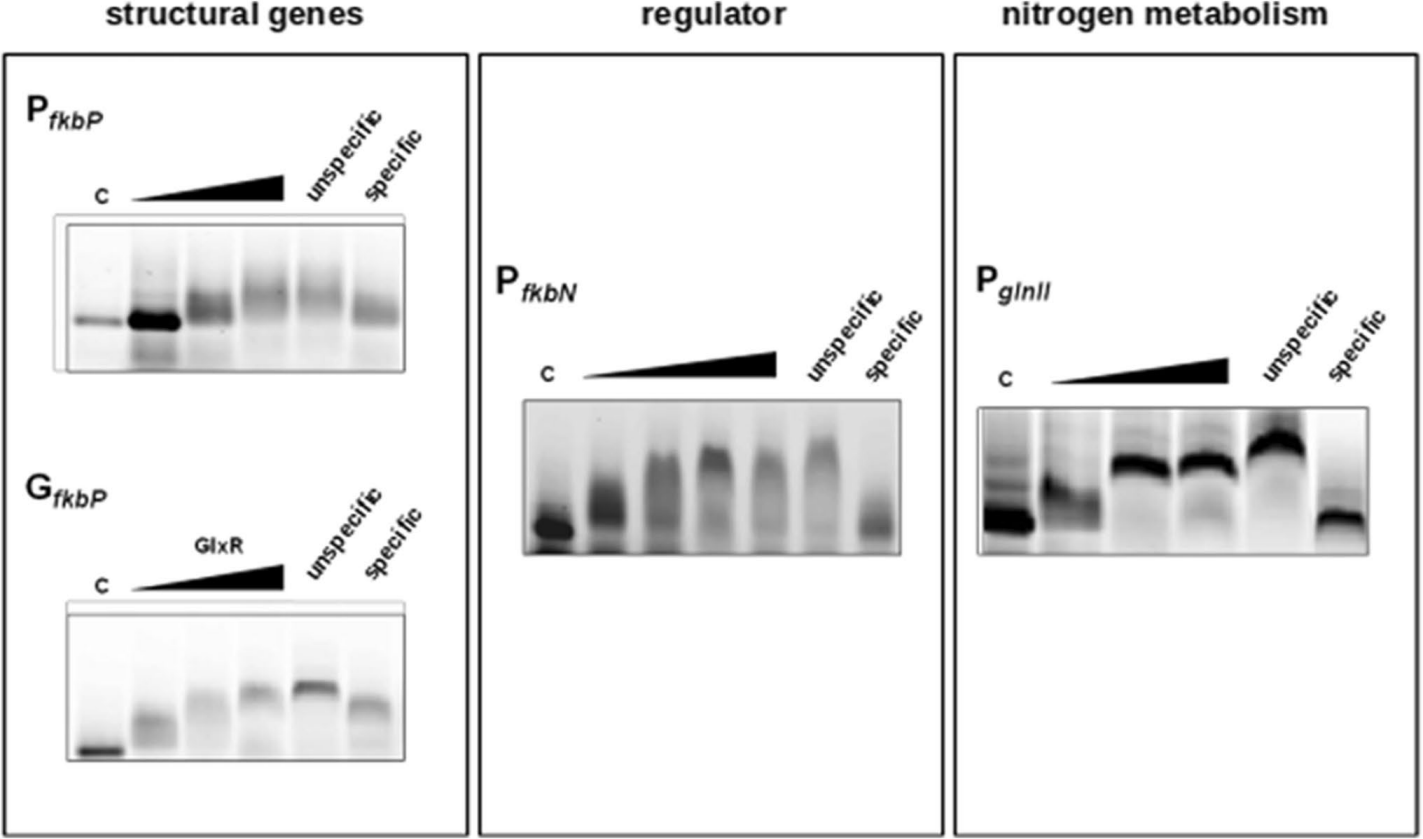
EMSA analysis of purified His-GlxR. Binding to the 160 bp promoter area sequence of selected genes: *fkbP*, *fkbN*, *glnII*. Samples without protein (c), with the 300 × excess of unspecific DNA and with the specific, unlabeled DNA were used as controls. His-GlxR was added from lower to higher concentration (black arrow)

Therefore, the promoter regions of the following genes were selected for subsequent EMSAs: *fkbP* coding for the NRPS in FK-506 synthesis; *fkbN* a regulatory gene encoding a positive regulator; and g*lnII* coding for the glutamine synthetase II. Promoter fragments (160 bp) of the target genes were amplified and labeled with Cy5. The labeled DNA fragments were then used for EMSA studies with GlxR. Thereby, band shifts of all DNA fragments with His-GlxR were observed (Fig. 6). These data indicate that the predicted Crp binding sequences of *S. tsukubaensis* can be recognized by the global regulator GlxR, a close homolog of Crp from *S. tsukubaensis*.

### Evaluation of the FK-506 and FK-520 production in the *S. tsukubaensis crp* overexpression strain in BioLector microbioreactor and 3L fermentor

**Fig. 7:**
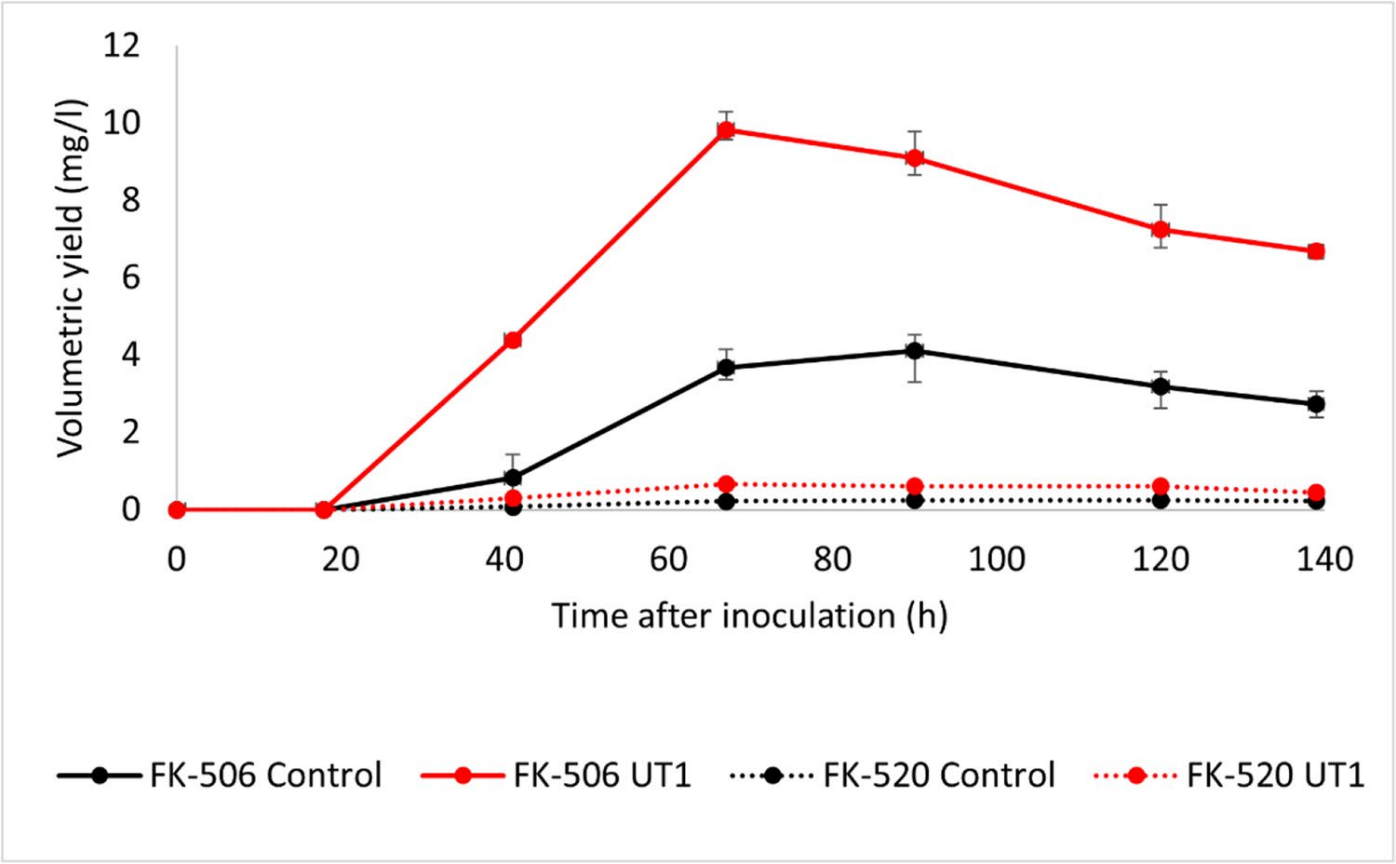
Average volumetric yield (mg/l) of FK-506 and FK-520 by the *S. tsukubaensis* UT1 strain (red line) and the control *S. tsukubaensis* (pRM4) (black line) (three replica of each condition). The strains were cultivated in a BioLector microfermentation system using 0.5 × MG-2.5 mM medium. The error bars represent the maximum and minimum values at each sampling point

In order to prove the observed positive effect of the *crp* overexpression on the FK-506 production, the *S. tsukubaensis* UT1 strain carrying an additional copy of *crp* and the control *S. tsukubaensis* (pRM4) strain were cultivated in a BioLector microreactor. The samples were analyzed for production of FK-506 and the FK-506 derivative ascomycin (FK-520). The results showed a significantly increased volumetric yield (mg/l) of both FK-506 and FK-520 in cultures with the *S. tsukubaensis* UT1 strain, compared to the control *S. tsukubaensis* (pRM4) (Fig. 7).

**Fig. 8:**
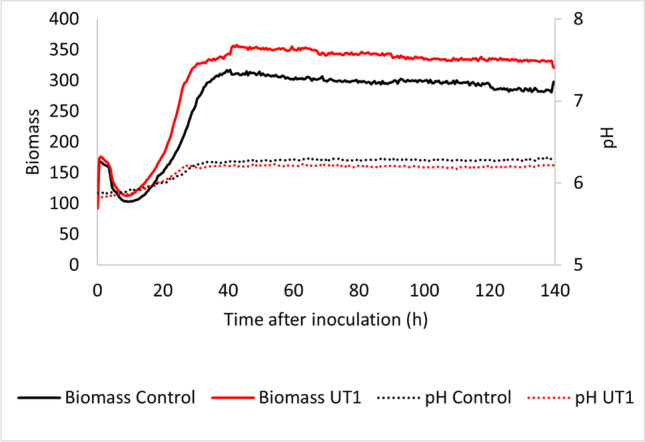
Average biomass (measured as scattered light intensity) and pH obtained in microfermentation of the *S. tsukubaensis* UT1 (red line) and the control *S. tsukubaensis* (pRM4) (black line) (three replica of each strain)

The biomass (measured as scattered light intensities) and the pH are given in Fig. 8. *S. tsukubaensis* UT1 obtained more biomass, although the growth rates of the two strains were similar. The maximum volumetric yields of FK-506 and FK-520 were obtained already after 67 h for *S. tsukubaensis* UT1, 9.8 mg/l and 0.67 mg/l, whereas after 90 h for the control strain, 4.1 mg/l and 0.25 mg/l, respectively.

**Fig. 9:**
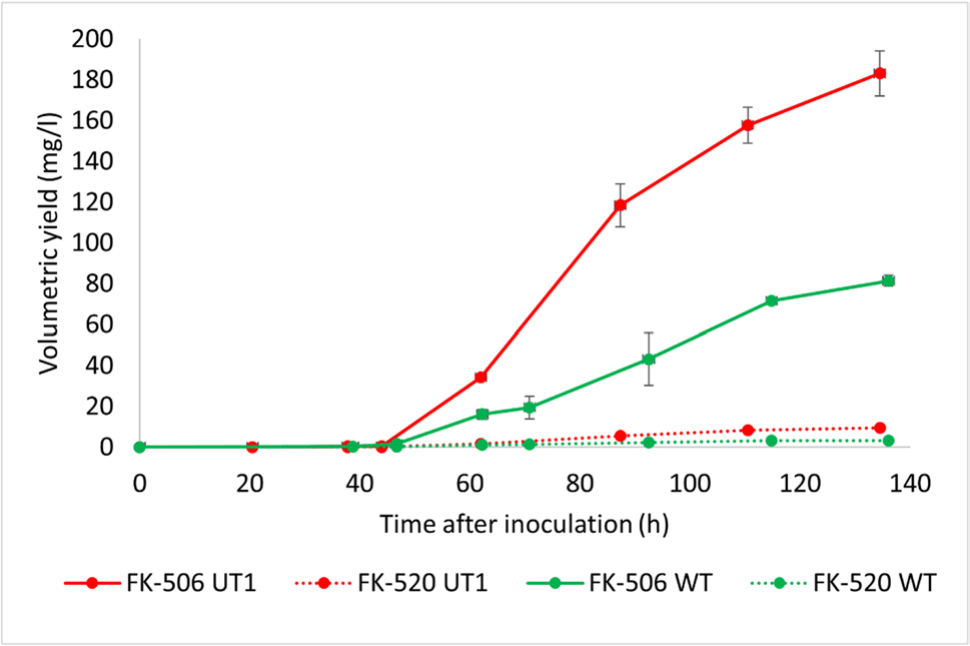
The average volumetric yield of FK-506 and FK-520 obtained in 3-L fermenation with 2 × MG-2.5 mM medium of cultures with *S. tsukubaensis* UT1 carrying an additional copy of *crp* and the WT *S. tsukubaensis* NRRL18488, two replica of each strain. The error bars show the maximum and minimum values at each sampling point

**Fig. 10:**
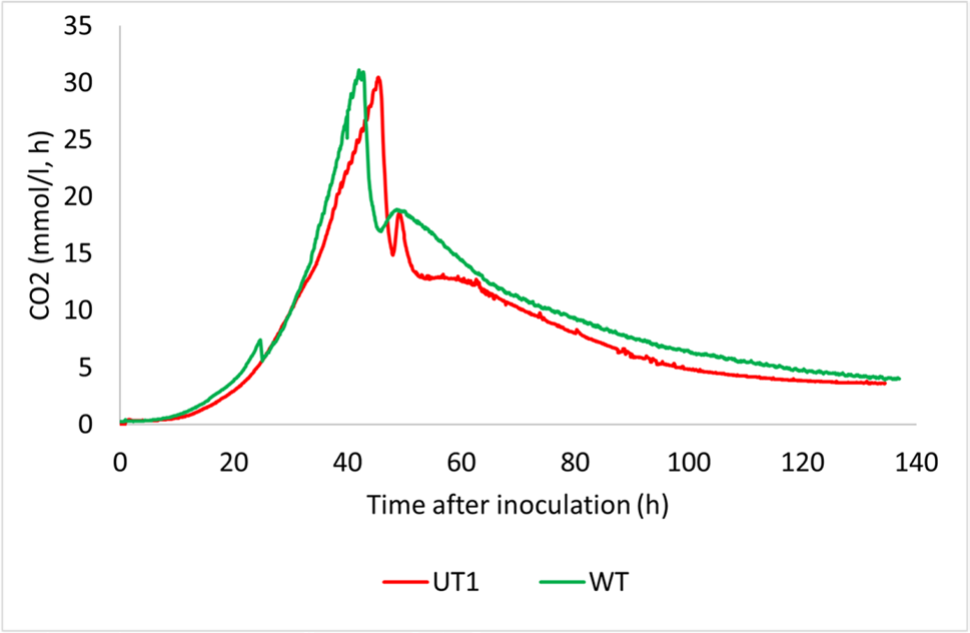
The carbon evolution rate (CER) obtained in 3L fermentation with 2 × MG-2.5 mM medium with *S. tsukubaensis* UT1 carrying an additional copy of *crp* and the WT *S. tsukubaensis* NRRL18488. The figure shows the average of two parallel cultivations

Further, *S. tsukubaensis* UT1 and the control strain *S. tsukubaensis* NRRL18488 (WT) were cultivated in fermentors (3-L) under controlled conditions in MG-2.5 mM medium. For both strains, the maximum volumetric yield of FK-506 was obtained after 91 h, and the volumetric yields of FK-506 for the two strains were 60.2 mg/l (*S. tsukubaensis* UT1) and 49.8 mg/l (WT) (Fig. S5). The cell mass was higher in *S. tsukubaensis* UT1 cultivation, and the specific production for *S. tsukubaensis* WT and *S. tsukubaensis* UT1 were 8.0 and 8.2 mg/g, respectively. Also, the carbon evolution rate (mmol CO2/l, h) was higher for the *S. tsukubaensis* UT1 when cultivated in MG-2.5 mM medium (Fig. S6). In shake flask cultivations, the medium concentration of MG-2.5 mM cannot be increased due to pH changes when glutamate is consumed. However, pH control in a microreactor allows it for more concentrated media with possibly higher volumetric yields. *S. tsukubaensis* UT1 and the *S. tsukubaensis* WT were cultivated in 2 × MG-2.5 medium with pH control. The average volumetric yield (two parallels) of FK-506 was 183.1 mg/l (172 mg/l, 194 mg/l) for *S. tsukubaensis* UT1 and 81.3 mg/l (79 mg/l and 84 mg/l) for the WT (Fig. 9). Production continued until 140 h of cultivation for both *S. tsukubaensis* UT1 and the WT. The volumetric yield of FK-506 in samples harvested from the WT after 164 h and 184 h were 73 mg/l and 82 mg/l, respectively (data not shown), showing that the product can be harvested after 140 h. Overexpression of Crp in 2 × MG-2.5 mM medium did not affect the carbon evolution rates (Fig. 10), which strongly indicate that the slight difference between the two strains is the production and not the growth rate and cell mass.

## Discussion

### Crp directly influences the expression of FK-506 biosynthetic genes

In this work, the global regulator Crp was investigated as a target for the enhancement of FK-506 production. In the FK-506 biosynthesis gene cluster, numerous Crp binding sequences in the promoter areas were identified. Analyses indicated that a Crp-mediated regulation of the FK-506 gene cluster takes place, since the overexpression of *crp* led to a rapid increase of the expression of genes from the FK-506 biosynthetic cluster and an improvement of FK-506 production. Recently also other studies reported that Crp can be used for enhancement of secondary metabolite production (Liu et al. 2020; Wu et al. 2021a). Furthermore, our results are in agreement with further studies, which demonstrated that the overexpression of other global regulators like *afsR* and *afsR2* resulted in stimulation of secondary metabolite production in *Streptomyces* (Ishizuka et al. 1992; Vögtli et al. 1994; Floriano and Bibb 1996).

Further investigations aimed to show the binding of the Crp protein to putative Crp-DNA binding sequences. However, no interaction of Crp with promoter regions of putative target genes was observed, Even the addition of the cAMP in various concentrations (up to 500 µM) did not result in an effect in EMSA assays. Previous attempts to perform Crp binding analysis (Derouaux et al. 2004a, b; Gao et al. 2012) were not successful as well. Interestingly, the unusual pI (isoelectric point) value of the *Streptomyces* Crp (e.g., 5.79 in *S. tsukubaensis*) is unusually low compared to Crp homologs in other bacteria, which may constitute a major obstacle for the implementation of EMSA studies. For example, Crp in *E. coli* is a basic protein with an isoelectric point of 9.12 (Harman 2001) and GlxR in *C. glutamicum* has a pI value of 6.60 (Kim et al. 2004).

Since GlxR has a Crp-equivalent function in corynebacteria and since both *Corynebacterium* and *Streptomyces* belong to the same genera of GC-rich bacteria, GlxR was included in our studies. GlxR from *C. glutamicum* shows great similarity with Crp and both display similar binding sequences (Kohl et al. 2008; Gao et al. 2012). Our results demonstrated that GlxR could bind the target sequences in the FK-506 biosynthetic gene cluster and nitrogen metabolism. This enabled to confirm the binding of the Crp homolog to identified putative Crp binding sequences in *S. tsukubaensis* indirectly, demonstrating an influence of this kind of regulators on genes from the FK-506 cluster.

### Cultivation under controlled conditions demonstrates increased production of FK-506 as an effect of Crp overexpression

The positive effect on FK-506 production by overexpression of Crp was demonstrated in cultivation experiments using a media previously optimized for FK-506 production (Martínez-Castro et al. 2013). In contrast to previous shake flask studies, we here demonstrated how a step-wise increase of medium component concentrations and fermentation scale impacted the production. Well plate microfermentation with 1.2 ml medium/well using 0.5 × MG-2.5 mM resulted in a volumetric yield of FK-506 that was ~ 1/3 of the yield obtained in 1 × medium (Martínez-Castro et al. 2013). Even in this microscale screening format, a doubling of the volumetric yield of FK-506 was evident under Crp overexpression. The production profile of the UT1 strain cultivated in 0.5 × MG-2.5 mM medium (Fig. 7), in 1 × MG-2.5 mM medium (Fig. S5), and in 2 × MG-2.5 mM medium (Fig. 9) demonstrated that the volumetric yield of the mutant was significantly higher early in the production phase. This can be explained from the initial high expression level of the biosynthetic gene cluster demonstrated (Fig. 4), and the observation is also in accordance with the initial production study (Fig. S3). The highest volumetric yield of FK-506 was achieved in 2 × MG-2.5 mM medium with controlled pH. Under these condition, *S. tsukubaensis* UT1 yielded 2.3 × more than the *S. tsukubaensis* WT reaching a maximum volumetric yield of 183.1 mg/l FK-506. Increasing the concentration of the medium further is challenging due to the solubility of starch used as carbon source.

The formation of FK-520 by most known producers, along with FK-506, can very seriously complicate the procedure for isolating and purifying the FK-506 to pharmacopoeial quality. Increase in FK-506 production unfortunately did not lead to a loss of production of the unwanted FK-520. Therefore, further work is needed to construct an FK-506 producer that no longer co-produces FK-520.

### Crp controls not only pathway-specific biosynthetic genes but also the expression of genes involved in basic functions

The regulation exerted by Crp may be a measure to link the expression of the FK506 biosynthetic genes with the physiological status of the cell. This happens on different levels: on the one hand, directly by activating the expression of biosynthetic genes or by controlling genes of the primary metabolism and on the other hand, indirectly by governing genes encoding pathway-specific regulators.

In this work, influence of Crp on the regulatory gene *fkbN* was observed, which encodes FkbN (Mo et al. 2012; Santos et al. 2012; Goranovič et al. 2012), a positive regulator of the FK-506 biosynthetic genes. Enhanced expression of *fkbN* in the *S. tsukubaensis* strain UT1 carrying an additional copy of *crp* suggested that Crp may cause an interaction with the specific regulatory gene resulting in the influence on FK-506 biosynthesis. Also, the regulatory gene *allN* appeared to be influenced by Crp overexpression of *crp* could enhance the expression of *aIIN* over the time. But since the deletion of the *allN* gene had no effect on the FK-506 production (Goranovič et al. 2012), the function of AllN in FK-506 biosynthesis remained obscure.

Interestingly, no influence of Crp on the expression of the *fkbR* gene was observed. The reason may be that FkbR plays a minor role in the regulation of the FK-506 biosynthesis in *S. tsukubaensis* and only the higher-level, direct activator FkbN can be influenced by Crp.

Crp targets in *E. coli* include specific regulators of carbon metabolism and regulators of nitrogen metabolism, such as ArgR, GlnG, and RpoS (Shimada et al. 2011). In this work not only participation of Crp on the expression of regulatory and biosynthetic genes of FK-506 production was found but also a direct role in the control of genes of the nitrogen metabolism was observed. This is in agreement with rather old observations that the nitrogen content in the cell represents a signal for the production of antibiotics or secondary metabolites (Aharonowitz 1980). The genes *amtB*,* glnK*,* glnD*,* gltB*,* glnII*, and *glnA* were selected based on the presence of Crp binding sequences in front of them and on the knowledge of their possible involvement in antibiotic production (Vega-Palas et al. 1992; Mao et al. 2007; Waldvogel et al. 2011). The microarray data from *S. coelicolor* showed that the expression of *glnK* and *amtB* was significantly upregulated under nitrogen-limiting conditions (Nieselt et al. 2010; Lewis et al. 2011). In *Streptomyces venezuelae*, the transcription of the homologous genes of *amtB*, *glnD*, *glnK*, and *glnII* was induced (Pullan et al. 2011). The expression profile of the central genes of nitrogen metabolism was influenced by Crp, namely *gltB*, *glnA*, and *glnII*. These genes code for the enzymes of the GOGAT pathway (Reuther and Wohlleben 2007). This assimilation pathway is active under limiting conditions and thus mirrors a state of stress in the cell. These results suggest that not all genes of primary nitrogen metabolism are induced for the synthesis of secondary metabolite precursors but only selected genes. Thus, Crp can have a modulating role in the primary metabolism in *S. tsukubaensis* influencing indirectly the FK-506 production.

The studies carried out within the scope of this work revealed new aspects of the Crp regulation in *Streptomycetes*. They confirm the hypothesis that the global regulatory protein Crp controls and coordinates versatile cellular functions not only in the well investigated *E. coli* and *B. subtilis*, but also in *Streptomyces*. This relationship represents a novel starting point for a FK-506 production optimization that can be used as a general optimization strategy for the production of secondary metabolites.

## Supplementary Information

Below is the link to the electronic supplementary material.

Supplementary file1 (PDF 427 KB)

## Data Availability

All data generated or analyzed during this study are included in this published article and its supplementary information files.
