# Supplementary material for: Optimization of FK-506 production in Streptomyces tsukubaensis by modulation of Crp-mediated regulation
Source: Appl Microbiol Biotechnol. 2023 Mar 23;107(9):2871–86. doi: 10.1007/s00253-023-12473-9 (PMC10033298; doi:10.1007/s00253-023-12473-9)
Supplement: Supplementary file 1 — Supplementary file1 (PDF 427 KB) [file 253_2023_12473_MOESM1_ESM.pdf]

Supplementary materials

Journal: Applied Microbiology and Biotechnology

Title: Optimization of FK-506 production in *Streptomyces tsukubaensis* by modulation of Crp-mediated regulation

Authors: Susann Schulz[1,2,*,], Håvard Sletta[3,**], Kristin Degnes[3], Sergii Krysenko[1,4,5], Alicia Williams[1], Silje Malene Olsen[3], Kai Vernstad[3], Agnieszka Mitulski[1,4,***], Wolfgang Wohlleben[1,4]**

1) Department of Microbiology and Biotechnology, Interfaculty Institute of Microbiology and Infection Medicine Tübingen (IMIT), University of Tübingen, Auf der Morgenstelle 28, 72076 Tübingen, Germany
2) Present address: Novartis AG, Stein, Switzerland
3) SINTEF Industry, Department of Biotechnology and Nanomedicine, Richard Birkelands vei 3, Trondheim, Norway
4) Cluster of Excellence 'Controlling Microbes to Fight Infections', University of Tübingen, Auf der Morgenstelle 28, 72076 Tübingen, Germany
5) Present address: Valent BioSciences, 1910 Innovation Wy Suite 100, Libertyville, IL 60048, United States of America

Corresponding Author: Wolfgang Wohlleben, Department of Microbiology and Biotechnology, Interfaculty Institute of Microbiology and Infection Medicine Tübingen (IMIT), University of Tübingen, Auf der Morgenstelle 28, 72076 Tübingen, Germany. Tel.: +49 07071 29-76944, Fax: (07071) 29-5979, eMail: wolfgang.wohlleben@biotech.uni-tuebingen.de.

Corresponding Author: Håvard Sletta, Biotechnology and Nanomedicine, SINTEF Industry, Richard Birkelands vei 3, Trondheim, Norway, Tel.: +47 915 44 429, eMail: havard.sletta@sintef.no

*née: Susann Kocadinc

**joint co-author

***formerly Bera

Supplementary

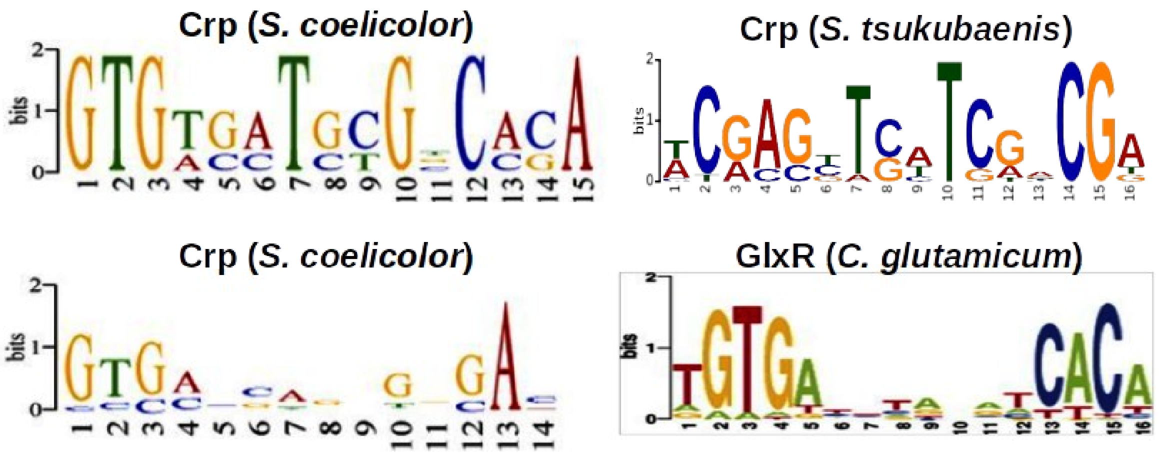

Figure S1. Complete analyzed motifs of Crp binding sequences in *S. coelicolor, S. tsukubaensis* and *C. glutamicum.* Comparison of the strong conserved [GTG(N)$_6$GNCAC] (top panel, left) and less conserved [GTG(N)$_6$GNGAN] (bottom panel, left) binding sequences of Crp in *S. coelicolor* (left) (Gao et al. 2012) with predicted binding sequences of Crp in *S. tsukubaensis* [NCGAG(N)$_6$GNCGA] (top panel, right) (this work) and of GlxR in *C. glutamicum* [NGTG(N)$_8$CACN] (bottom panel, right) (Kohl et al. 2008).

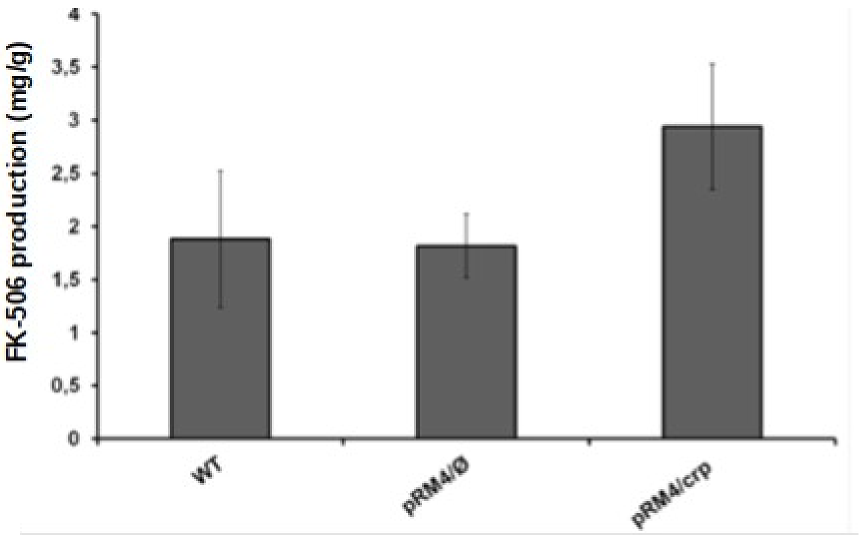

Figure S2. FK-506 yield in different *S. tsukubaensis* strains. Calculation of the mean value of FK-506 production. pRM4 stays for the integrative plasmid without an inserted gene. PRM4/*crp* - integration of an additional copy of the gene on the integrative plasmid pRM4 encoding *crp* – a global regulator Crp. n=3.

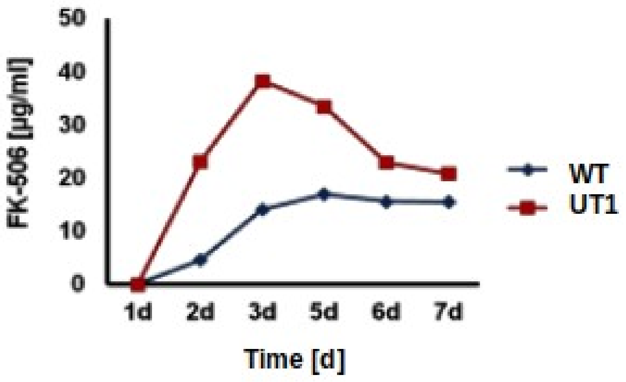

Figure S3. HPLC analyses during the FK-506 production phase in the *S. tsukubaensis* wild type (WT) and the strain with the introduced *crp* overexpression plasmid (UT1).

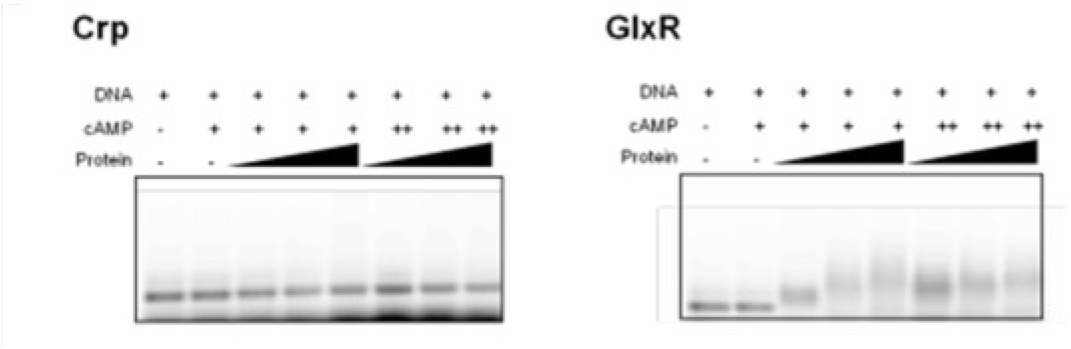

Figure S4. EMSA analysis with purified Crp or GlxR and the 160 bp promoter sequence from *cya* on 2% agarose gel. The cAMP concentration added was variable up to 500 µM (increasing from - to ++). 0.5-2 µg StrepII-Crp or His-GlxR (increasing arrow) was added.

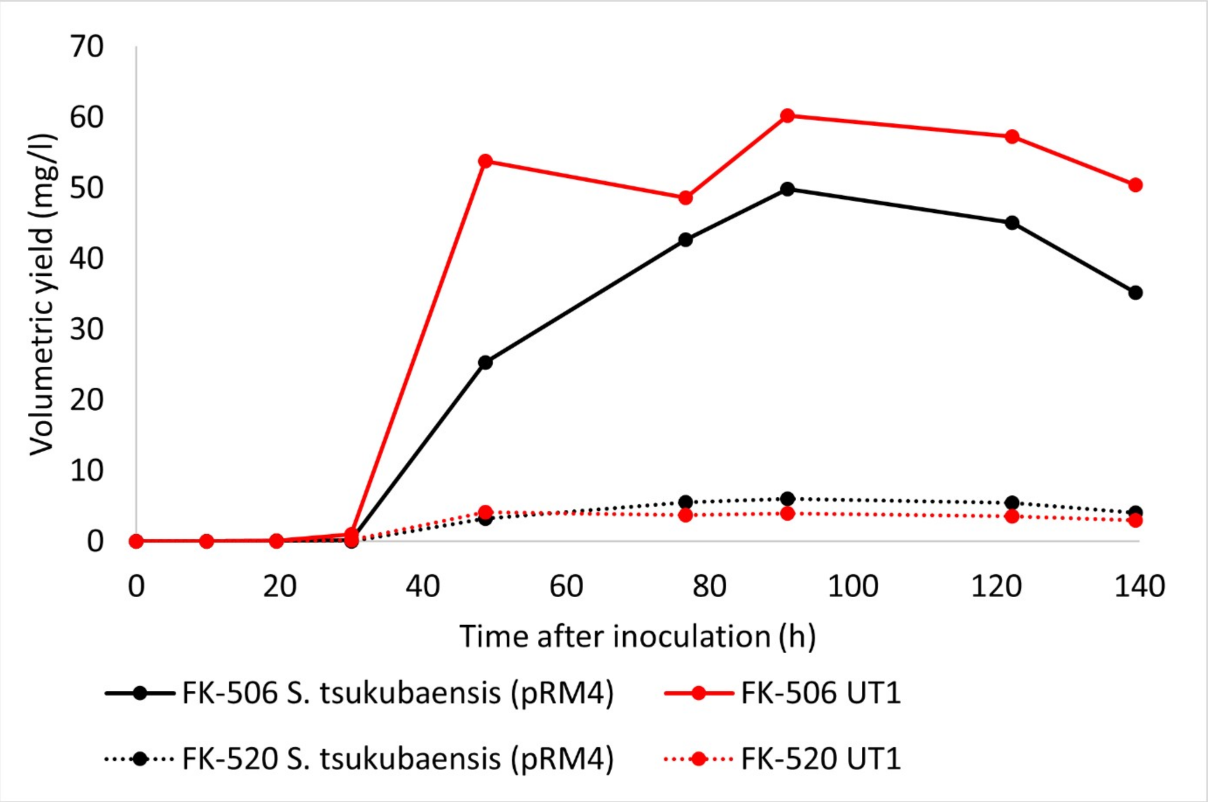

Figure S5: The volumetric yield of FK-506 and FK-520 obtained in 3L fermentation with 1 x MG-2.5 medium with the *S. tsukubaensis* UT1 strain and the control *S. tsukubaensis* (pRM4).

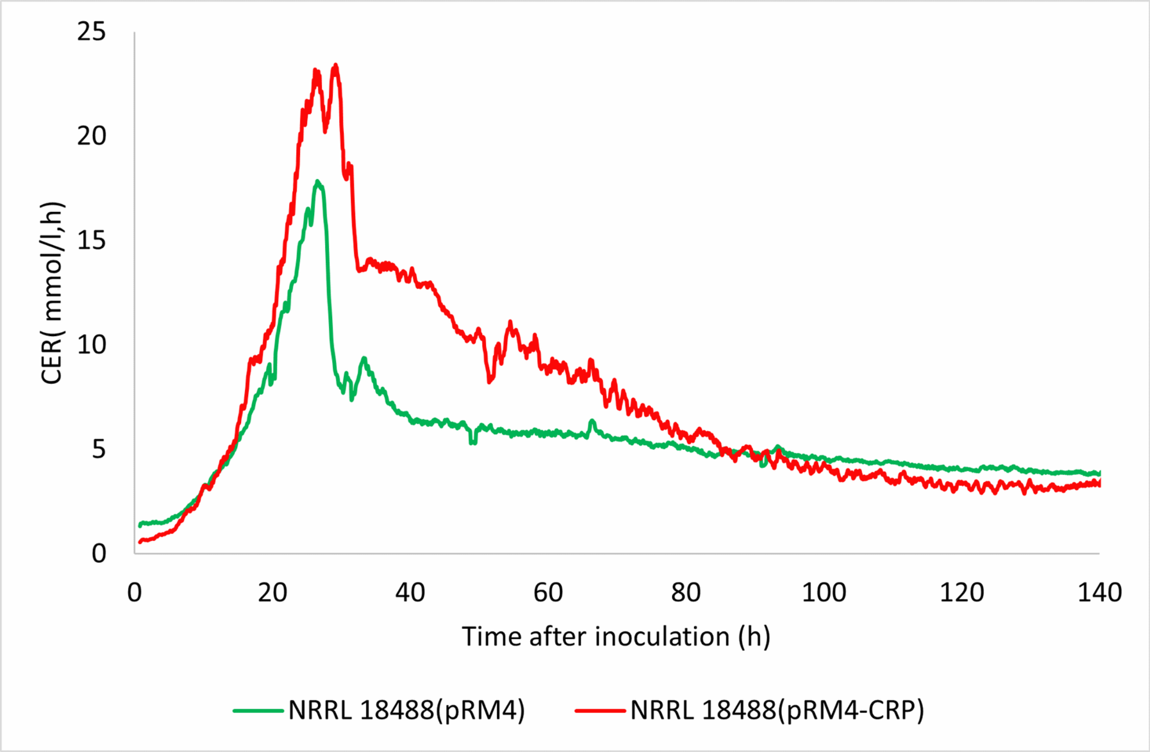

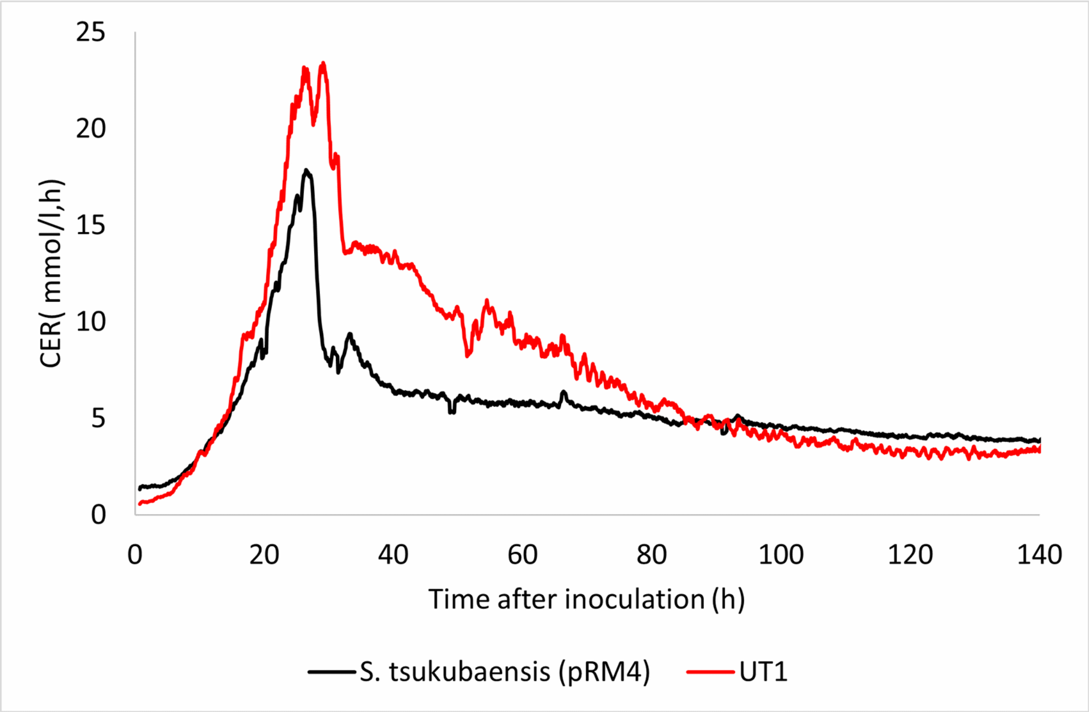

Figure S6. The carbon evolution rate (CER) obtained in 3L fermentation with 1 x MG-2.5 medium with the *S. tsukubaensis* UT1 strain and the control *S. tsukubaensis* (pRM4).

Table S1. Identified *in silico* Crp binding sequences in selected genes of *S. tsukubaensis*.

Gene promoter region	Crp binding sequence

fkbB	GTGAGTGTCGGCGAC
fkbO	GTGAGTGTCGGCGAC
fkbC	CGCCTCATCCCTTCC
fkbL	GTGTTCTTCGCCGC
crp	GTGCGTGCTGCGA
cya	GTGAGCTGGGGCACA
glnA	GTGGCTGACCGCA
glnII	GTGGGAAATCGCC
gltB	GTGGAACCCGGCG

Table S2. Strains and plasmids used in this study.

Strains/Plasmids	Genotype	Reference
Streptomyces tsukubaensis NRRL18488	STP1, STP2	Martinez-Castro et al. 2011, 2013
Streptomyces tsukubaensis UT1	Derivative of *S. tsukubaensis* WT for *crp* overexpression; *crp* introduced on the pRM4 plasmid; Apr^R	This work
S. tsukubaensis (pRM4)	Derivative of *S. tsukubensis* WT with an empty plasmid pRM4 introduced;	This work

	Apr^R	
E. coli NovaBlue	recA1, endA1, gyrA96, thi-1, *hsdR17* (rK12 - ,mK12 +) *supE44*, *relA1*, lac [F', *proAB*, *lacIq*, *lacZ*ΔM15, Tn10] (Tet^R)	Novagen
E. coli Rosetta 2(DE3) pLysS	Derivate of BL21, pRARE2: 7 rare tR-NAs; rare *E. coli* codons: arginine (AGA, AGG, CGA) glycine (GGA), isoleucine (AUA), leucine (CUA), pro-line (CCC)	Novagen
E. coli ET12567/ pUZ8002	Methylation deficient strain *E. coli* with pUZ8002, F-, *dam-* 13::Tn*9*, *dcm*-6, *hsdM*, *hsdR*, *lacY*1, Cam^R, Kan^R	MacNeil et al. 1992
E. coli ET12567/pUB307	Methylation deficient strain *E. coli* with pUB307, F-, *dam*-13::Tn*9*, *dcm*-6, *hsdM*, *hsdR*, *lacY*1, Cam^R, Kan^R, Tet^R	MacNeil et al. 1992
E. coli ET12567/ pUZ8002-pRM4-*glxR*	pRM4-derivative with *glxR;* Apr^R, Cam^R; Kan^R	This work
pRM4	pSET152*ermEp** with artificial RBS, Apr^R	Menges et al. 2007
pRM4-*crp*	pRM4-derivative with *crp;* Apr^R	This work
pRM4-*glxR*	pRM4-derivative with *glxR*; Apr^R	This work
pYT9	pJOE2775-derivative, expression of *glnR* with StrepII-Tag, Amp^R	Tiffert et al. 2008
pYT9-*glnR*-StrepII	pJOE2775-derivative with PCR ampli-fied Strep–*glnR* cloned *Nde*I-*Hin*dIII	Tiffert et al. 2008
pYT9-*crp*StrepII	pYT9-derivative, *glnR* replaced by *crp*	This work

	for the production and purification of StrepII-Crp	
pET-*glxR*	pET30-derivative for expression of the *glxR* gene with the His-Tag	This work

Table S3. Oligonucleotides used in this study

Oligonucleotides	Sequences 5´-3´	Reference
RTPF	GTACGACGGTGTTCATGACG	This work
RTPR	TCTGAGGACCTGCAGCAGTA	This work
RT-AIIN-F	CAGAGGTTTCCAGGCTCAGG	This work
RT-AIIN-R	TCCAGAATACGAGGTGGGTCT	This work
RTB1F	GGAAGTCGAGTCGCTGTACG	This work
RTB1R	ACAGAACGCTCGTCTGGATT	This work
RTB2F	CACCTCGTCGACCACTACG	This work
RTB2R	TCCTGGTCCTTGGTGAGTTC	This work
RTOF	GCTCTACACGCGGGTCTTC	This work
RTOR	GCAGGGTTCTCGATGTTGAT	This work
RT_fkbN_F	TCCTCGTCACCTGCAATGAC	This work
RT_fkbN_R	GTCGGCTATCTCCCTGTTCG	This work
RT_gltB_F	CTACAAGGGCATGCTCACCA	This work

RT_gltB_R	GTACAGACCGGGAAGATGCG	This work
RTLF	TTTCGTACAGTCCGCACAAC	This work
RTLR	CGGGTCGATATCGCTGAC	This work
RT_fkbR_F	GAGTGCAGGTACGTCTCACC	This work
RT_fkbR_R	ATGTCCAGTTGCAGGCCC	This work
RT-CRPST-F	TCAAACTCCACCGCACCTC	This work
RT-CRPST-R	CATCGGAGAAGACCAGGTCG	This work
RT_cya_F	GCAGATCGACTCCTTCTTGG	This work
RT_cya_R	GTTCACCGAGCTCCTCCTCT	This work
RT_glnII_F	ACATCATGACCTTCCGGCTG	This work
RT_glnII_R	AGTTCACCCACTGGTTGGTG	This work
hrdB_1	AGCAAGGGCTTCGTCCTGTC	This work
hrdB_2	TTGCCCTCCTGGATCAGGTC	This work
pL-C31f	CCCACAGCTGGAGGCCGTGG	This work
pL-C31r	CAGGGCGAGCAATTCCGAGA	This work
pR-C31f	CAGAGCAGGATTCCCGTTGAG	This work
pR-C31r	CCCTTCATCATGATGGACCAG	This work
